# Temperature Increase during Different Post Space Preparation Systems: An In Vitro Study 

**Published:** 2011-08-15

**Authors:** Kiumars Nazari Moghadam, Shahriar Shahab, Soghra Shirvani, Ali Kazemi

**Affiliations:** 1 *Department of Endodontics, Dental School, Shahed University of Medical Sciences, Tehran, Iran*; 2 *Department of Oral and Maxillofacial Radiology, Dental School, Shahed University of Medical Sciences, Tehran, Iran*; 3 *Dental Student, Shahed University of Medical Sciences, Tehran, Iran*

**Keywords:** Periodontal Ligament, Post Technique, Root Canal Therapy, Root Canal Preparation Tooth Fracture

## Abstract

**INTRODUCTION:** The purpose of this study was to evaluate external root surface temperature rise during post space preparation using LA Axxess bur, Beefill pack System, and Peeso Reamer drill.

**MATERIALS AND METHODS:** The distal canals of forty-five extracted human permanent mandibular first molars were instrumented in crown-apical manner and obturated with lateral condensation technique. Teeth were then randomly divided into three groups according to post space preparation technique including: group 1. LA Axxess bur (Sybronendo Co., CA, USA), group 2 Beefill pack System (VD W Co., Munich, Germany) and group 3 Peeso Reamer drill (Mani Co., Tochigi-ken, Japan). Temperature was measured by means of digital thermometer MT-405 (Comercio Co., Sao Paulo, Brazil) which was installed on the root surfaces. Data was collected and submitted to one-way ANOVA and Post hoc analysis.

**RESULTS:** Root surface temperatures were found to be significantly higher (7.3±2.7 *vs.* 4.3±2.1 and 4±2.4,) in samples of Beefill pack System compared with the two other groups (P<0.02).

**CONCLUSION:** Using Beefill pack System during post space preparation may be potentially hazardous for periodontal tissues.

## INTRODUCTION

Temperature increase on the root surface during post space preparation and subsequent damage to the root cementum, alveolar bone and periodontal ligament have been reported in many *in vitro *and *in vivo* studies (1-4). Temperature increase of 10^º^C above body temperature for >1min can be destructive for bone tissue (1-4). Moreover, temperature increase up to 53^º^C for 1min resulted in either blood flow cessation or sluggishness of vessels (5).

Rotary instruments, ultrasonics, heat, heat/hand instruments, hand files, chemical technique, paper point with chemicals have been used to prepare post space (1-4). Rotary and heat may increase the temperature on the root surface to a critical level (6). Previous studies comparing different rotary methods revealed that surface temperatures were significantly higher for peeso reamer drills (7). Temperature increase after using system B during Thermafill retreatment has the range of 26.7-46^º^C which can potentially damage periodontal tissues.

Therefore, using system B for post space preparation may be potentially hazardous for periodontal tissues (8). Beefill pack System is a new device which has been professionally designed for three-dimensional warm vertical compaction. This system has different pluggers with length markings for down packing, and removal of gutta-percha during post space preparation (9). LA Axxess bur with non-cutting tip, parabolic interface in different taper has revolutionized coronal third preparation; this system does not jeopardized surrounding tissues during gutta-percha removal (10).

**Figure1:**
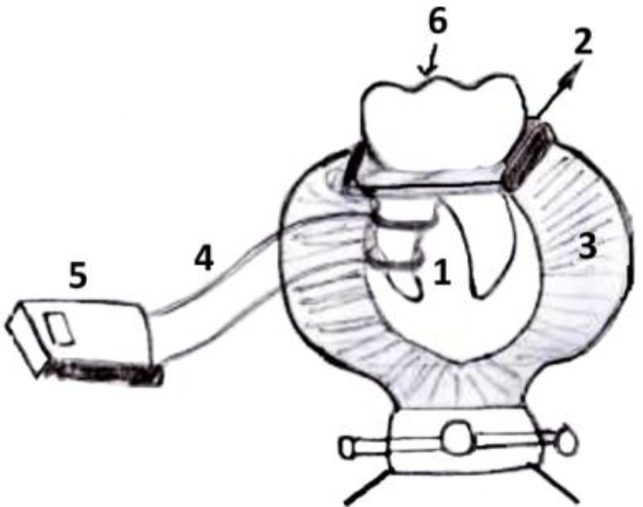
schematic view: 1-leads, 2-ortho acrylic resin, 3-vise, 4-wire, 5-Digital thermometer, 6-Tooth

LA Axxess bur used for cervical root canal preparation for 5sec have been found to have safe temperature increases (11). The aim of this *in vitro* study was to compare temperature rise on the root surface during post space preparation using LA Axxess bur with those of Beefill pack System and peeso reamer drills.

## MATERIALS AND METHODS

In this experimental study, forty-five extracted human permanent mandibular first molars with at least 19mm length**,** apical curvature of 20 degree (Schneider method), mature apex and uncalcified canals were used. The length of all roots was adjusted to approximately 17mm from the coronal reference point to the apex by means of cutting above the cemento-enamel junction; they were prepared using FlexMaster rotary system (VDW, Munich, Germany) up to 30/0.06 in crown-down technique. All the prepared canals were obturated laterally with gutta-percha cone and ADSeal root canal sealer (Cheongwon-gun, Chungbuk, Korea). Dentine thickness at different thirds was matched by means of Cone Beam Computed Tomography (CBCT) New tom VG (Quantitative Radiology, Verona, Italy), so that the three groups were similar. All 45 samples were randomly divided into three groups of 15 teeth each. About 11mm of coronal gutta-percha was removed by either peeso reamer drills #2, #3 (Mani Co., Tochigi-ken, Japan) (group 1), LA Axxess Bur (Sybronendo Co., CA, US) with 20/0.06, then with 35/0.06 (group 2), or Beefill pack System (VD W Co., Munich, Germany) (group 3) with 30/0.04 tip so that 6mm of the apical gutta-percha remained in the canals.

The teeth were placed in an ortho-acrylic resin; they were fixed by a coronal attachment to facilitate gutta-percha removal and placement of the Digital thermometer MT-405 lead (two K-Type) (Comercio Co., Sao Paulo, Brazil) on the external surface of the root (Figure 1). In order to record the temperature change at the apical-middle and coronal-middle thirds of the roots, two leads were positioned and connected to digital thermometer by wetting root site with conductive gel. Radiographs were taken to ensure the adequacy of the gutta-percha removal after post space preparation. Each instrument was used inside the root canal for 10sec. Any temperature change on external surface of root temperature was recorded. Data was collected and submitted to one-way ANOVA (significance level of 5%) and Post hoc analysis.

**Table 1:** Mean and standard deviation of temperature increase (˚C) among studied groups

**Group**	**Mean±SD**	**Min**	**Max**
LA Axxess bur	4.02±2.42	0.5	7.4
Peeso reamer	4.36±2.19	1.4	7.9
Beefill pack	7.34±2.70	1.5	11.3

## RESULTS

Root surface temperature increase was found significantly higher (P<0.02) for Beefill pack System in comparison with two others instruments. Post hoc analysis by Tukey HSD showed that there was significant difference between the mean temperature increase of Beefill pack System with peeso reamer drill (P value=0.01), and Beefill pack System with LA Axxess bur (P value=0.003).

## DISCUSSION

This study showed that using Beefill pack System created more temperature increase than peeso reamer drill and LA Axxess bur. There was significant difference between peeso reamer bur with Beefill pack System and LA Axxess bur with Beefill pack System. On the basis of this study, the use of Beefill pack System may be potentially hazardous because of the temperature increase higher than 10˚C from the basal level (body temperature) which causes irreversible damage to the surrounding tissues of root (1-4).

Greater temperature increase in the mandibular incisors compared to maxillary anteriors during obturation with system B has been shown (12); the highest temperature rise occurred 5 to 6mm from the apex (12,13). Researcher has shown that. System B at various temperature setting never reached the critical 10^º^C rise with any temperature setting or tip configuration (14). Floren *et al.* evaluated temperature increase using System B at various temperature settings from 250˚C to 600˚C; the highest temperature was recorded at a distance of 5mm from the root apex; this was the only site with exceeded temperature over 10˚C (12). This different results presented in these studies might be attributed to differences in methodology (size of samples, statistical analysis, and the technique of measuring the temperature). The temperature increase of root surface is multi-factorial and dependent on the root's dentin thickness, the extent of contact between instrument and canal wall, intermittently or continuously usage. Ultrasonics have also been studied with regard to post space removal temperature increase (15,16). Root surfaces on thin/thick roots undergoing ultrasonic vibration on their cemented posts were tested; no significant relationship between temperature rise and dentin thickness was found (16). The role of gutta-percha thickness and root canal sealer as insulator may interfere with thermal conductivity.

In such *in vitro *study impediments such as the quantity of blood flow around the tooth, the tooth position/site in the arch and alternating irrigant usage are unknown. Moreover, the time taken to return (elapsed time) to normal temperature was not checked. The use of LA Axxess bur during post space preparation decreases the risk of thermally induced damage to the periodontium.

## CONCLUSION

Using Beefill pack system during post space preparation may be potentially hazardous for periodontal tissues.
